# Diabetes mellitus and its associated risk factors in patients with human immunodeficiency virus on anti-retroviral therapy at referral hospitals of Northwest Ethiopia

**DOI:** 10.1186/s13098-020-00527-1

**Published:** 2020-03-04

**Authors:** Alemu Gebrie, Bekele Tesfaye, Tensae Gebru, Fentahun Adane, Worku Abie, Mekonnen Sisay

**Affiliations:** 1 Department of Biomedical Science, School of Medicine, Debre Markos University, P.O. Box 269, Debre Markos, Ethiopia; 2 Department of Nursing, College of Health Sciences, Debre Markos University, Debre Markos, Ethiopia; 3 Department of Biochemistry, School of Medicine, College of Health Sciences, Addis Ababa University, Addis Ababa, Ethiopia; 4 Department of Pharmacology and Toxicology, School of Pharmacy, College of Health and Medical Sciences, Haramaya University, P.O. Box 235, Harar, Ethiopia

**Keywords:** Diabetes mellitus, Risk factors, HIV/AIDS, Ethiopia

## Abstract

**Background:**

The use of highly active anti- retroviral therapy (HAART) as well as human immunodeficiency virus (HIV) per se have been shown to be related with diabetes among patients living with HIV. There is limited evidence on the prevalence of diabetes among HIV-infected patients in developing countries like Ethiopia. Therefore, the aim of this study is to determine the prevalence of diabetes among patients living with HIV/AIDS at referral hospitals of Northwest Ethiopia.

**Materials and methods:**

a hospital based cross-sectional study was conducted at referral hospitals of Northwest Ethiopia between February 2019 and April 2019. Using WHO stepwise approach, sociodemographic, behavioral and clinical data were collected from 407 included adult patients. Simple random sampling methods was used to select the study participants. Lipid profiles, fasting blood sugar as well as anthropometric indicators were also measured. SPSS version 25 was used for analysis of data; bivariate and multivariate binary logistic regression analysis was performed.

**Result:**

From a total of 415 patients living with HIV deemed eligible for inclusion, 407 with complete data were included in the final analysis giving a response rate of 98%. From 407 study subjects included in the analysis, 161 (39.6%) were men. The prevalence of diabetes mellitus was found to be 8.8% (95% CI 6.05, 11.55). Multivariate logistic regression analysis revealed that age [AOR (95% CI) 1.04 (1.001,1.084), p < 0.05], educational status [AOR (95% CI) 6.27 (1.72, 22.85), p < 0.05, diploma; AOR (95% CI) 9.64 (2.57, 36.12), p < 0.05, degree and above], triglyceride level [AOR (95% CI) 1.007 (1.003, 1.010), p < 0.01] have shown statistically significant association with odds of diabetes mellitus.

**Conclusion:**

The prevalence of diabetes was notably high in patients living with HIV/AIDS. Factors such as increased age, educational status and higher level of serum triglyceride were found to contribute to this high prevalence of diabetes.

## Background

As of the end of 2018, about 37.9 million people globally were living with HIV. By the end of June 2019, 24.5 million people were accessing antiretroviral therapy [1]. In Ethiopia, about 690, 000 people were living with HIV with adult prevalence rate of about 1%, and 65% of people living with HIV were on treatment [2, 3]. A systematic review revealed the prevalence of diabetes in Ethiopian population ranging from 2.0 to 6.5% with low (2%) in smaller rural areas [4]. HIV and noncommunicable diseases (NCDs) have become public health concerns in our globe. Studies indicated that HIV/AIDS and NCDs have linkage which may be direct effects of HIV or indirectly by the drug regimens [5–9]. Plethora of evidences have shown an increased risk of metabolic abnormalities like dysregulation of glucose metabolism in HIV infected patients [10–13]. Although blood glucose levels tend to be increased in antiretroviral‐naïve HIV‐infected individuals by mechanisms that remain to be understood, evidence supports that HAART could intensify this effect by altering lipid metabolism and increasing insulin resistance [14, 15].

The use HAART as well as HIV per se have been shown to be related with diabetes among patients living with HIV (PLWHIV). Because HIV-infected patients are living longer with HAART and better HIV care, an increase in NCDs in this population is inevitable. The era of highly active antiretroviral therapy has been purported by an increase in non‐AIDS–defining illnesses. Compared with HIV-negative subjects, HIV-infected individuals on HAART have a higher prevalence as well as earlier onset of diabetes mellitus [7, 16]. Studies have found various risk factors related with the occurrence of NCDs in HIV patients. Factors such as gender, physical inactivity, smoking, alcohol use, family history of diabetes mellitus, CD4 count, viral load, duration of HIV infection and old age have been shown to be responsible for the high prevalence of diabetes in such HIV-infected population [14, 16, 17]. In particular, the development of diabetes mellitus in people living with HIV has also been shown to be contributed by the use of certain specific medications that are component of combination HAART regimens [18]. A meta-analysis conducted including 41 studies revealed that the risk of diabetes mellitus in PLWHIV was about four times more as compared with treatment-naïve individuals, and has concluded HAART to be the single most important predictor of diabetes mellitus in people living with HIV [19].

The co-existence of diabetes in HIV-infected patients could complicate HIV infection management. In patients living with HIV/AIDS, diabetes mellitus has been revealed to be related with a higher risk of hospitalization, and adverse renal and cardiovascular outcomes such as advanced renal disease stage consequently decreasing life expectancy and being vicious cycle to the already- high costs of care for these patients [20–22]. Findings related to NCDs in HIV-positive patients is beneficial to influence various HIV programs and policies. In addition, researches on the area are worth undertaking to identify the risk factors associated with the problems so that relevant measures can be forwarded to reduce the morbidity and mortality of patients with HIV/AIDS. Despite the fact that diabetes and other metabolic abnormalities are impending problems in low income countries like Ethiopia with notable HIV prevalence, limited evidence exists on the disease burden of diabetes in the HIV-infected individuals in the study area. Therefore, the aim of this study was to determine the prevalence of diabetes mellitus and its associated factors among HIV infected patients in referral hospitals of Northwest Ethiopia.

## Materials and methods

### Study area and setting

The study was conducted at Felege Hiwot Referral Hospital (FHRH) and Debre Markos Referral Hospital (DMRH) in Northwest Ethiopia. The hospitals are among the few referral hospitals in Northwest Ethiopia. FHRH, which is found at Bahir Dar Town, mainly gives services to people from West Gojjam zone, Awi Zone and Bahir Dar Town, the capital of Amhara National Regional State. Similarly, DMRH, found at Debre Markos Town which the capital of East Gojjam Zone, is the only referral hospital in East Gojjam administrative zone. The referral hospitals serve millions of people in the surrounding catchment area. The two referral hospitals currently serve a total of about 10,213 patients (3788 patients at DMRH and 6425 patients at FHRH) living with HIV/AIDS. Activities in the referral hospitals include: HIV testing and counseling, CD4 count and viral load tests to monitor the prognosis of the treatment, antiretroviral drugs dispensation, health education to enhance treatment adherence and management of defaulters, and monitoring of opportunistic infections.

### Study design and period

Institution based cross-sectional study was conducted from February 2019 and April 2019.

### Study population

The study population were adult HIV-positive people receiving HAART from the ART clinics in the referral hospitals during the study period.

### Inclusion exclusion criteria

PLWHIV with ≥ 18 years old age, on HAART for at least 6 months, and those who signed informed consent were included in the study. On the other hand, pregnant females, those with diabetes and/or hypertension before HAART, and patients with serious illness who are unable to give appropriate information were excluded from the study.

### Sample size determination and sampling procedure

A sample size of 415 was taken using a single population proportion formula:

$$n = \frac{{z_{{{\raise0.7ex\hbox{$a$} \!\mathord{\left/ {\vphantom {a 2}}\right.\kern-0pt} \!\lower0.7ex\hbox{$2$}}}}^{2} pq}}{{d^{2} }}$$

The assumptions are: 50% prevalence (p = 1− q = 0.05) of diabetes mellitus among HIV infected patients in the study area since there is no previous study in the area, 95% level of confidence level (z_α/2_ = 1.96), 5% of marginal error (d = 0.05), and 8% for anticipated non-response. Sample was proportionally taken from the referral hospitals based on the patient load, 154 from DMRH and 261 from FHRH. At the ART clinic in the referral hospitals, daily patient flow was accessed from the data logbook so as to select the study participants. The participants were selected using simple random sampling technique, by lottery method.

### Data collection procedure

#### Study variables

Diabetes mellitus among patients living with HIV/AIDS was the outcome variable in this study. Patient associated factors like sex, age, occupational and economic status and marital status; HIV related such as viral load, co-morbid state, HAART duration, WHO stage of the disease, duration HIV infection, opportunistic infections; behavioral factors including: drug use history, lifestyle of the patients, and treatment linked factors such as type of HAART regimens, concomitant drug therapy, duration of HAART were predictor variables of this study.

### Sociodemographic, clinical and anthropometric data

Sociodemographic, behavioral and clinical data were collected by using structured questionnaire adapted from WHO step wise approach to chronic disease risk factor surveillance [23, 24] via patient interview and chart review. Anthropometric data (weight, height, waist circumference, and hip circumference) as well as blood pressure were also collected. Procedures for collection of data anthropometric (weight, height, waist circumference) and blood pressure were standardized prior to be used for data collection. Waist circumference (WC) were measured with a flexible inelastic tape placed on the midpoint between the lower rib margin and the iliac crest in a perpendicular plane to the long axis of the body. Height was determined without shoes using a portable stadiometer. Weight was measured using a Tanita scale; patients were fully dressed, without heavy clothing or shoes. Body mass index (BMI) was calculated by dividing the weight of the patients in kilograms to the square of their height in meters. All the measurements were carried out under the standard operating procedure by two trained and experienced nurses under supervision for the quality of the data. Blood pressure (BP) was measured as a mean of the last two of three measurements with the Omron Automatic Inflation Blood Pressure Monitor taken at intervals longer than 2 min after the patient was sitting for at least 30 min.

#### Blood sample collection and laboratory analysis

Approximately 5 mL blood sample was withdrawn from each study participant’s cubital vein on the next appointment after fasting for 12 h using vacutainer blood collection system to determine lipid profiles and fasting blood sugar (FBS). Laboratory analysis of sample was done by experienced laboratory technologists under laboratory standard operating procedures. Blood samples were analyzed for FBS level using by glucose oxidase-phenol amino phenazone (GOD-PAP) method, and lipid profile (HDL, LDL, Triglyceride and total cholesterol) determined using ABX Pentra 400 using an automated clinical chemistry analyzer machine.

#### Operational definition and terms

As per the WHO criteria, FBS ≥ 126 mg/dL (fasting defined as no caloric intake for at least 8 h) [25], and/or an already diagnosed diabetes defines diabetes mellitus in this study. BMI was classified as normal/underweight: BMI < 25 kg/m^2^, overweight: BMI 25–29.9 kg/m^2^ and obese: BMI ≥ 30 kg/m^2^. Study subjects were considered smokers, if they do smoke at least one cigarette within the last 12 months, and alcohol consumers, if they drink at least twice within a week of any alcoholic stuff. Abnormal waist circumferences of male and female are ≥ 94 and ≥ 80 cm, respectively [26]. Abnormal lipid status was defined as per the USA National Cholesterol Education Program: total cholesterol ≥ 200 mg/dL, HDL-cholesterol < 40 mg/dL, LDL-cholesterol (≥ 130 mg/dL) and triglycerides ≥ 150 mg/dL [27]. Comorbidity state is defined as those concurrent diseases other than opportunistic infections such as cardiovascular diseases, asthma, kidney diseases etc., and another drug treatment includes those drugs other than HAART for the treatment of the comorbidities.

### Data quality control

High emphasis was given by close supervision of the process to assure data quality. The questionnaire was standardized and pretested to tailor it to the hospitals set up at 5% (n = 20) of similar subjects at Finote Selam Hospital before the actual data collection to rectify any doubt or difficulty appeared. The data collectors were nominated by their experience and expertise in the area. The collected data was checked for completeness, clarity and consistency before any analysis. In addition, all the laboratory analyses were conducted by experienced laboratory technologists under standard operating procedures.

### Data processing, analysis and presentation

After checking the completeness and consistency, data were entered and analyzed using Epi-Data version 3.1 and the Statistical package for Social Science (SPSS) version 25, respectively. Socio-demographic, epidemiological, clinical, and laboratory values were presented and described using frequency distribution tables, and figures. While continuous variables were reported as mean ± SD, categorical variables were presented as percentages. Logistic regression analysis was used to determine the associations factors with diabetes mellitus. Variables with *p* value less than 0.25 in bivariate logistic regression analysis were declared eligible for multivariate logistic regression analysis. Hosmer and Lemeshow goodness of fit test carried out before conducting the multiple binary logistic regressions. p-value less than 0.05 in the multivariate analysis was taken as cut-off value for significant association at 95% confidence interval.

## Result

### Socio‑demographic and behavioral characteristics of participants

From a total of 415 patients living with HIV deemed eligible for inclusion, 407 with complete data were included in the final analysis giving a response rate of 98%. From 407 study subjects included in the analysis, 161 (39.6%) were men. The average age of the study subjects was 38.6 with a standard deviation of 10.3 years. About 28.7% of the participants were not engaged in any formal education, and about half of the participants (53.8%) were not married. Two-third of the participants were not employed in any work and live in urban area with most of them having low monthly income. Whereas about 11.8% of the participants had history of alcohol drinking habit, only 2.5% of them did smoke cigarette and majority of them (87%) were not engaged in regular physical activity (Table 1).

**Table 1:** Socio-demographic and behavioral characteristic of people living with HIV/AIDS receiving care at referral hospitals of Northwest Ethiopia, 2019 (n = 407)

Variables	Category	Frequency (%)
Age (years)	Mean ± SD	38.64 ± 10.29
Sex	Male	161 (39.6)
	Female	246 (60.4)
Educational status	No education	117 (28.7)
	Elementary	148 (36.4)
	High school	71 (17.4)
	Diploma	42 (10.3)
	Degree/above	29 (7.1)
Marital status	Never married	44 (10.8)
	Currently married	188 (46.2)
	Divorced	78 (19.2)
	Widowed	63 (15.5)
	Separated	34 (8.4)
Occupation	Gov employee	71 (17.4)
	NG employee	22 (5.4)
	Self-employed	186 (45.7)
	Non-paid	27 (6.6)
	Student	17 (4.2)
	Farmer	34 (8.4)
	Homemaker	50 (12.3)
Income (ETB)	< 2000	222 (54.5)
	2000–5000	150 (36.9)
	> 5000	35 (8.6)
Residence	Urban	277 (68.1)
	Rural	130 (31.9)
Alcohol consumption	Yes	48 (11.8)
	No	359 (88.2)
Smoking status	Yes	10 (2.5)
	No	397 (97.5)
Physical exercise	Yes	53 (13)
	No	354 (87)

*SD* standard deviation, *NG* non-governmental, *ETB* Ethiopian Birr

### Diabetes and clinical as well as anthropometric related characteristics

**Fig. 1:**
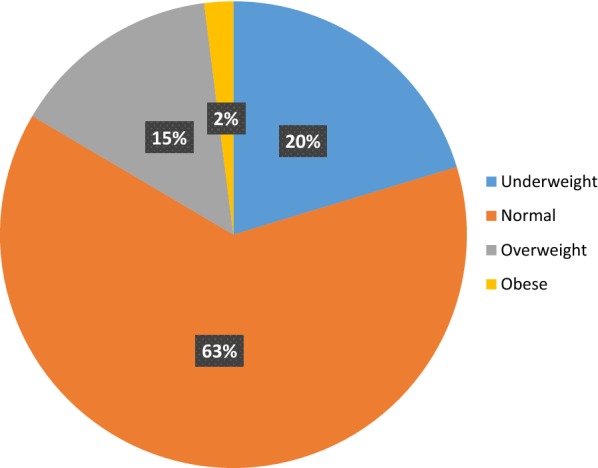
BMI of patients living with HIV at referral hospitals of Northwest Ethiopia, 2019

In the present study, it has been revealed that the prevalence of diabetes was 8.8% (95% CI 6.05, 11.55) (Table 2). About 16.5% of the patients were either overweight or obese with an average BMI of 21.5 ± 3.73 (Table 2, Fig. 1). Regarding to the regional distribution of fat, two-fifth of the participants had waist circumference above the cut-off value and about 75% of the patients had elevated waist to hip ratio which is a better indicator of regional fat distribution. Only 9.3% of the participants had viral load more than 1000 and 13% of them had comorbidity or opportunistic infection. In addition, about 12% of the HIV patients used drugs and substances other than HAART. Moreover, almost all the patients were in WHO stage-I, 71.1% of the patients with HIV duration of more than 5 years and the most frequent HAART regiment was TDF/3TC/EFV (Table 2).

**Table 2:** Clinical and anthropometric related characteristics of people living with HIV/AIDS receiving care at referral hospitals of Northwest Ethiopia, 2019 (n = 407)

Variable	Category	Frequency (%)
Body Mass Index(kg/m^2^)	Mean ± SD	21.5 ± 3.73
Waist circumference	High	168 (41.3)
	Normal	239 (58.7)
Waist to hip ratio	High	305 (74.9)
	Normal	102 (25.1)
Viral load	Less than 1000	369 (90.7)
	More than 1000	38 (9.3)
Comorbidity state	Yes	65 (16)
	No	342 (84)
WHO clinical stage	Stage I	403 (99.0)
	Stage II	4 (1.0)
Opportunistic Infection	Yes	15 (3.7)
	No	392 (96.3)
Another drug treatment	Yes	33 (8.1)
	No	374 (91.9)
Type of HAART regimen	TDF/3TC/EFV	186 (45.7)
	AZT/3TC/NVP	96 (23.6)
	TDF/3TC/NVP	42 (10.3)
	TDF/3TC/ATV/r	33 (8.1)
	AZT/3TC/EFV	14 (3.4)
	AZT/3TC/ATV/r	36 (8.8)
Another substance/drug use	Yes	13 (3.2)
	No	394 (96.8)
Duration of HIV/AIDS	< 5 years	77 (18.9)
	5–10 years	178 (43.7)
	> 10 years	152 (37.3
Diabetes mellitus	Yes	36 (8.8)
	No	371 (91.2)

### Factors associated with diabetes mellitus in HIV infected patients

In bivariate logistic regression analysis, age, educational status, monthly income, BMI, waist circumference, waist to hip ratio, triglyceride level, total cholesterol level, viral load, diastolic blood pressure and concomitant drug had p value < 0.25 and included in multivariate logistic regression analysis.

In the multivariate analysis, age was significantly associated with diabetes mellitus. For each year increase in age, the patients were 1.04 more likely to have diabetes, [AOR (95% CI) 1.04 (1.001,1.084), p < 0.05]. Patients who have diploma as their educational status were about 6.27 times more likely to have diabetes mellitus as compared to no formal education counterparts, [AOR (95% CI) 6.27 (1.72, 22.85), p < 0.05]. In addition, patients who have degree and above were about 9.64 times more likely to have diabetes mellitus as compared to patients with no formal education, [AOR (95% CI) 9.64 (2.57, 36.12), p < 0.05]. Moreover, serum level of triglyceride was found to have a statistically significant association with diabetes mellitus. For every unit increase in the level of triglyceride in HIV infected patients, 0.7% more likely to develop diabetes mellitus [AOR (95% CI) 1.007 (1.003, 1.010), p < 0.01] (Table 3).

**Table 3:** Bivariate and multivariate analyses of socio-demographic, anthropometric and clinical risk factors for diabetes mellitus of people living with HIV/AIDS receiving care at referral hospitals of Northwest Ethiopia, 2019 (n = 407)

Variable	Category	Diabetes mellitus		Bivariate analysis		Multivariate analysis	
		No	Yes	COR (95% CI)	p-value	AOR (95% CI)	p-value
Age				1.04 (0.01, 1.08)	0.008*	1.04 (1.001,1.084)	0.043*
Educational status	No education	112	5	1	–	1	–
	Elementary	139	9	1.45 (0.47, 4.45)	0.516	1.68 (0.52, 5.44)	0.386
	High school	63	8	2.84 (0.89, 9.07)	0.077	3.09 (0.840, 11.40)	0.090
	Diploma	35	7	4.48 (1.34, 15.01)	0.015*	6.27 (1.72, 22.85)	0.005*
	Degree/above	22	7	7.13 (2.07, 24.51)	0.002*	9.64 (2.57, 36.12)	0.001*
Monthly income	< 2000	205	17	1	–	1	–
	2000–5000	136	14	1.24 (0.59, 2.60)	0.567	0.71 (0.28, 1.82)	0.470
	> 5000	30	5	2.01 (0.69, 5.85)	0.200	0.58 (0.132, 2.44)	0.446
Body mass index				1.08 (0.99, 1.18)	0.067	1.12 (0.97, 1.29)	0.128
Waist circumference				1.05 (1.01, 1.09)	0.019*	0.98 (0.93, 1.03)	0.397
Waist to hip ratio				11.63 (2.65, 22.42)	0.014*	2.34 (0.25, 22.80)	0.172
Triglyceride				1.01 (1.004,1.010)	0.000*	1.007 (1.003,1.010)	0.000*
Diastolic BP				0.98 (0.94, 1.02)	0.246	0.965 (0.925, 1.006)	0.094
Total cholesterol				1.004 (0.998, 1.01)	0.227	1.00 (0.99, 1.006)	0.625
Viral load	< 1000	339	30	1	–	1	–
	> 1000	32	6	2.12 (0.82, 5.47)	0.121	2.48 (0.85, 7.20)	0.095
Concomitant drug	Yes	28	5	0.51 (0.18, 1.40)	0.191	2.56 (0.84, 7.87)	0.100
	No	343	31	1	–	1	–

## Discussion

There has been a scantiness of data on the potential burden of diabetes mellitus and other cardiometabolic factors among HIV patients in Africa particularly in Ethiopia. This study was aimed to determine the burden of diabetes mellitus, and identify factors associated with diabetes in HIV/AIDS patients in referral hospitals of Northwest Ethiopia.

One of the key findings of the present study was the relatively high prevalence of diabetes (8.8%) in the HIV-infected participants. The estimated prevalence in this study is significantly higher than the 2019 IDF report (ninth edition) of national prevalence (3.2%) of diabetes in Ethiopian adult (20–79 years) population [28] and the 2016 WHO report of the national prevalence (3.8%) of diabetes in the general population of Ethiopia [29]. It is also higher than the diabetes prevalence (6.8%) report of a community-based cross-sectional study conducted in 2019 in northeast Ethiopia [30] as well as a meta-analysis report (4.99%) for Ethiopia population [31]. The higher prevalence has been supported by different studies [12, 32–34] indicating that HIV increases the risk of developing a number of health problems [35]. In addition, the observed prevalence of diabetes is comparable to 8.8% prevalence report at ART clinic of Dessie Referral Hospital, northeast, Ethiopia [36], 8.5% prevalence reported at Jimma University Specialized Hospital, Southwest, Ethiopia [37] and 8% from Wolayta Sodo Hospital, Southern Ethiopia [38]. However, the prevalence in this study is slightly higher than those reported at Tikur Anbessa Specialized Hospital in Addis Ababa, Ethiopia (6.9%) [39] and University of Gondar Hospital, Northwest Ethiopia (5.1%) [40]. In addition, our report was higher than the reports of those studies carried out in other countries, including Uganda (4%) [41], Malawi (4.1% and 6.6%) [42, 43] Zambia (5.0%) [44], but it is lower than the report of Senegal (14.5%) [45] and Tanzania (18.0%) [46]. The observed difference could be pertaining to variations in the lifestyle and HAART regimens or because of differences in the age distribution of the study participants. The discrepancy could also be justified that it could be due to differences in the clinical, economical, anthropometric and socio-demographic characteristics of the study populations [47]. Although the care of cardiometabolic risks related to HIV may be difficult in Ethiopia because of limited healthcare resources, it can be noted that metabolic clinic should be part of HIV services more importantly as the older HIV population is increasing. Strategies are required to deal with progressive increase in diabetes [48].

Age, educational status and serum triglyceride level of patients were significantly associated with diabetes mellitus. In line with previous studies [8, 18, 36, 37, 45, 49], we have found a higher risk of diabetes mellitus among older patients with HIV. For every year increase in age, there is about 4% more chance of having diabetes in the patients. With increased survival of infected patients treated with combination HAART, a rise in diabetes incidence independent of HIV-related influences may occur with ageing of this population [50, 51]. In addition, educational status is significantly associated with diabetes mellitus. As educational level advances, the risk of having diabetes increases. This could be explained because of unfavorable life style and over weight as well as regional fat distribution of those educated ones as compared to those with no formal education. Relationship between educational attainment and diabetes related problems such as overweight may be inverse, direct, null, and U-shape. The association of educational attainment and overweight depends on the country’s level of development, such that positive relations are more common in less developed countries and inverse relations are more common in more developed countries [52–54]. Moreover, higher serum level of triglyceride was found to a risk factor for diabetes mellitus which is consistent with the studies done in Malaysia [55], Nigeria [56], and Australia [57]. This is due to increased free fatty acid flux secondary to insulin resistance and aggravated by increased inflammatory adipokines [58].

## Limitation

Owing to the cross-sectional design of the study, we are not able to determine temporal relationships between DM and the associated factors. Stronger study designs like prospective cohort studies are then indicated so as to identify the predictors of diabetes mellitus in the study subjects. The subjective nature of the self-reported response for some items might also be limited by recall bias. Moreover, environmental factors such as the impact of dietary factors have not been studied, and enrolment of HIV negative controls would have possibly provided a comparison of biochemical changes in the study subjects. The study being limited to only two health institutions, generalizability could also be affected. However, the study has provided some data to inform decision-makers to improve current care and management of HIV-infected persons on HAART.

## Conclusion and recommendation

The prevalence of diabetes was notably high in patients living with HIV/AIDS. Factors such as increased age, educational status and higher level of serum triglyceride were found to contribute to this high prevalence of diabetes. The findings highlight the need for routine screening and assessment of factors for diabetes in HIV-infected persons receiving combination HAART. In addition, in the view of the fact that PLWHIV are an expanding and aging population to address, a metabolic clinic may also be a good choice to improve the clinical service.

## Data Availability

The original data of this study could be available for the third body only up on author’s request.

## Abbreviations

IDF: International Diabetes Federation
AOR: Adjusted odds ratio
HAART: Highly active anti-retro viral therapy
WC: Waist circumference
FBS: Fasting blood sugar
HDL: High density lipoprotein
LDL: Low density lipoprotein
TG: Triglyceride
TC: Total cholesterol
WHO: World Health Organization
NCDs: Non-communicable diseases
HIV/AIDS: Human immunodeficiency virus/acquired immunodeficiency syndrome
PLWHIV: People living with HIV

## References

[1] UNAIDS. Global HIV & AIDS statistics—2019 fact sheet. http://www.unaids.org/en/resources/fact-sheet.

[2] UNAIDS/country/Ethiopia: https://www.unaids.org/en/regionscountries/countries/ethiopia.

[3] EDHS. Ethiopia Demographic and Health Survey, 2016 HIV prevalence report: Central Statistical Authority; Addis Ababa, Ethiopia, and Rockville, Maryland, USA; CSA and ICF; 2016.

[4] Bishu KG, Jenkins C, Yebyo HG, Atsbha M, Wubayehu T, Gebregziabher M (2019). Diabetes in Ethiopia: a systematic review of prevalence, risk factors, complications, and cost. Obesity Medicine.

[5] Medina-Torne S, Ganesan A, Barahona I, Crum-Cianflone NF (2012). Hypertension is common among HIV-infected persons, but not associated with HAART. J Int Assoc Phys AIDS Care.

[6] Nüesch R, Wang Q, Elzi L, Bernasconi E, Weber R, Cavassini M, Vernazza P, Thurnheer MC, Calmy A, Battegay M (2013). Risk of cardiovascular events and blood pressure control in hypertensive HIV-infected patients: Swiss HIV Cohort Study (SHCS). JAIDS.

[7] Brown TT, Cole SR, Li X, Kingsley LA, Palella FJ, Riddler SA, Visscher BR, Margolick JB, Dobs AS (2005). Antiretroviral therapy and the prevalence and incidence of diabetes mellitus in the multicenter AIDS cohort study. Arch Intern Med.

[8] Ledergerber B, Furrer H, Rickenbach M, Lehmann R, Elzi L, Hirschel B, Cavassini M, Bernasconi E, Schmid P, Egger M (2007). Factors associated with the incidence of type 2 diabetes mellitus in HIV-infected participants in the Swiss HIV Cohort Study. Clin Infect Dis.

[9] Grinspoon S, Carr A (2005). Cardiovascular risk and body-fat abnormalities in HIV-infected adults. N Engl J Med.

[10] Larson R, Capili B, Eckert-Norton M, Colagreco JP, Anastasi JK (2006). Disorders of glucose metabolism in the context of human immunodeficiency virus infection. J Am Acad Nurse Pract.

[11] Gallagher DM (2007). Current clinical issues impacting the lives of patients living with HIV/AIDS. J Assoc Nurses AIDS Care.

[12] Wand H, Calmy A, Carey DL, Samaras K, Carr A, Law MG, Cooper DA, Emery S, Committee ITIC (2007). Metabolic syndrome, cardiovascular disease and type 2 diabetes mellitus after initiation of antiretroviral therapy in HIV infection. Aids.

[13] Nigatu T (2012). Integration of HIV and noncommunicable diseases in health care delivery in low-and middle-income countries. Preve Chronic Dis.

[14] Kalra S, Kalra B, Agrawal N, Unnikrishnan A (2011). Understanding diabetes in patients with HIV/AIDS. Diabetol Metab Syndr.

[15] Dagogo-Jack S (2008). HIV therapy and diabetes risk. Diabetes Care.

[16] Hernandez-Romieu AC, Garg S, Rosenberg ES, Thompson-Paul AM, Skarbinski J (2017). Is diabetes prevalence higher among HIV-infected individuals compared with the general population? Evidence from MMP and NHANES 2009–2010. BMJ Open Diabetes Res Care.

[17] Monroe AK, Glesby MJ, Brown TT (2014). Diagnosing and managing diabetes in HIV-infected patients: current concepts. Clin Infect Dis.

[18] Capeau J, Bouteloup V, Katlama C, Bastard J-P, Guiyedi V, Salmon-Ceron D, Protopopescu C, Leport C, Raffi F, Chêne G (2012). Ten-year diabetes incidence in 1046 HIV-infected patients started on a combination antiretroviral treatment. Aids.

[19] Nduka CU, Stranges S, Kimani PK, Sarki AM, Uthman OA (2017). Is there sufficient evidence for a causal association between antiretroviral therapy and diabetes in HIV-infected patients? A meta-analysis. Diabetes/Metab Res Rev.

[20] Akgün KM, Gordon K, Pisani M, Fried T, McGinnis KA, Tate JP, Butt AA, Gibert CL, Huang L, Rodriguez-Barradas MC (2013). Risk factors for hospitalization and medical intensive care unit (MICU) admission among HIV infected Veterans. J Acquir Immune Defic Syndr.

[21] Medapalli R, Parikh CR, Gordon K, Brown ST, Butt AA, Gibert CL, Rimland D, Rodriguez-Barradas MC (2012). Chang C-CH, Justice AC: comorbid diabetes and the risk of progressive chronic kidney disease in HIV-infected adults: data from the Veterans Aging Cohort Study. J Acquir Immune Defic Syndr.

[22] Jotwani V, Li Y, Grunfeld C, Choi AI, Shlipak MG (2012). Risk factors for ESRD in HIV-infected individuals: traditional and HIV-related factors. Am J Kidney Dis.

[23] World Health Organization (2010). WHO STEPS instrument (core and expanded).

[24] World Health Organization (2012). WHO STEPS instrument.

[25] Organization WH: Definition and diagnosis of diabetes mellitus and intermediate hyperglycaemia: report of a WHO/IDF consultation. 2006.

[26] WHO (2011). Waist circumference and waist-hip ratio: report of a WHO Expert Consultation, Geneva, 8–11 December 2008.

[27] Detection NCEPEPo, Adults ToHBCi: Third report of the National Cholesterol Education Program (NCEP) Expert Panel on detection, evaluation, and treatment of high blood cholesterol in adults (Adult Treatment Panel III): International Medical Pub; 2002. 12485966

[28] IDF DIABETES ATLAS Ninth edition 2019.

[29] WHO (2016). Diabetes country profles.

[30] Endris T, Worede A, Asmelash D (2019). Prevalence of diabetes mellitus, prediabetes and its associated factors in Dessie Town, Northeast Ethiopia: a community-based study. Diabetes Metab Syndr Obes.

[31] Tesfaye B, Alebel A, Gebrie A, Zegeye A, Tesema C, Ferede A, Abera H, Alam K (2019). Prevalence of diabetes mellitus and its association with hypertension in Ethiopia: a systematic review and meta-analysis. Diabetes Res Clin Pract.

[32] Brown T, Cole S, Li X, Kingsley L, Palella F, Riddler S (2005). Antiretroviral therapy and the prevalence and incidence of diabetes mellitus in the multicenter AIDS cohort study. Arch Intern Med.

[33] Tien PC, Schneider MF, Cole SR, Levine AM, Cohen M, DeHovitz J, Young M, Justman JE (2007). Antiretroviral therapy exposure and incidence of diabetes mellitus in the women’s interagency HIV Study. Aids.

[34] Silva E, Bassichetto K, Lewi D (2009). Lipid profile, cardiovascular risk factors and metabolic syndrome in a group of AIDS patients. Arq Bras Cardiol.

[35] Ataro Z, Ashenafi W, Fayera J, Abdosh T (2018). Magnitude and associated factors of diabetes mellitus and hypertension among adult HIV-positive individuals receiving highly active antiretroviral therapy at Jugal hospital, Harar, Ethiopia. HIV/AIDS.

[36] Fiseha T, Belete AG (2019). Diabetes mellitus and its associated factors among human immunodeficiency virus-infected patients on anti-retroviral therapy in Northeast Ethiopia. BMC Res Notes.

[37] Mohammed AE, Shenkute TY, Gebisa WC (2015). Diabetes mellitus and risk factors in human immunodeficiency virus-infected individuals at Jimma University Specialized Hospital, Southwest Ethiopia. Diabetes Metab Synd Obes.

[38] Sachithananthan V, Loha E, Gose M (2013). Prevalence of diabetes mellitus, hypertension and lipodystrophy in HAART receiving HIV patients in Southern Ethiopia. Int STD Res Rev.

[39] Feleke Y, Fekade D, Mezegebu Y (2012). Prevalence of highly active antiretroviral therapy associated metabolic abnormalities and lipodystrophy in HIV infected patients. Ethiop Med J.

[40] Abebe SM, Getachew A, Fasika S, Bayisa M, Demisse AG, Mesfin N (2016). Diabetes mellitus among HIV-infected individuals in follow-up care at University of Gondar Hospital, Northwest Ethiopia. BMJ Open.

[41] Omech B, Sempa J, Castelnuovo B, Opio K, Otim M, Mayanja-Kizza H, Colebunders R, Manabe YC (2012). Prevalence of HIV-associated metabolic abnormalities among patients taking first-line antiretroviral therapy in Uganda. Isrn Aids..

[42] Divala OH, Amberbir A, Ismail Z, Beyene T, Garone D, Pfaff C, Singano V, Akello H, Joshua M, Nyirenda MJ (2016). The burden of hypertension, diabetes mellitus, and cardiovascular risk factors among adult Malawians in HIV care: consequences for integrated services. BMC Public Health.

[43] Rücker SCM, Tayea A, Bitilinyu-Bangoh J, Bermudez-Aza EH, Salumu L, Quiles IA, Szumilin E, Chirwa Z, Rick F, Maman D (2018). High rates of hypertension, diabetes, elevated low-density lipoprotein cholesterol, and cardiovascular disease risk factors in HIV-infected patients in Malawi. AIDS.

[44] Shankalala P, Jacobs C, Bosomprah S, Vinikoor M, Katayamoyo P, Michelo C (2017). Risk factors for impaired fasting glucose or diabetes among HIV infected patients on ART in the Copperbelt Province of Zambia. J Diabetes Metab Disord.

[45] Diouf A, Cournil A, Ba-Fall K, Ngom-Guèye NF, Eymard-Duvernay S, Ndiaye I, Batista G, Guèye PM, Bâ PS, Taverne B (2012). Diabetes and hypertension among patients receiving antiretroviral treatment since 1998 in Senegal: prevalence and associated factors. Isrn Aids.

[46] Maganga E, Smart LR, Kalluvya S, Kataraihya JB, Saleh AM, Obeid L, Downs JA, Fitzgerald DW, Peck RN (2015). Glucose metabolism disorders, HIV and antiretroviral therapy among Tanzanian adults. PLoS ONE.

[47] Ngu RC, Choukem S-P, Dimala CA, Ngu JN, Monekosso GL (2018). Prevalence and determinants of selected cardio-metabolic risk factors among people living with HIV/AIDS and receiving care in the South West Regional Hospitals of Cameroon: a cross-sectional study. BMC Res Notes.

[48] Husain NE, Noor SK, Elmadhoun WM, Almobarak AO, Awadalla H, Woodward CL, Mital D, Ahmed MH (2017). Diabetes, metabolic syndrome and dyslipidemia in people living with HIV in Africa: re-emerging challenges not to be forgotten. HIV/AIDS.

[49] Tripathi A, Liese A, Jerrell J, Zhang J, Rizvi A, Albrecht H, Duffus W (2014). Incidence of diabetes mellitus in a population-based cohort of HIV-infected and non-HIV-infected persons: the impact of clinical and therapeutic factors over time. Diabet Med.

[50] Butt AA, McGinnis K, Rodriguez-Barradas MC, Crystal S, Simberkoff M, Goetz MB, Leaf D, Justice AC (2009). HIV infection and the risk of diabetes mellitus. AIDS.

[51] Samaras K (2012). The burden of diabetes and hyperlipidemia in treated HIV infection and approaches for cardiometabolic care. Curr HIV/AIDS Rep.

[52] Cohen AK, Rai M, Rehkopf DH, Abrams B (2013). Educational attainment and obesity: a systematic review. Obes Rev.

[53] Boissonnet C, Schargrodsky H, Pellegrini F, Macchia A, Marcet Champagne B, Wilson E, Tognoni G (2011). Educational inequalities in obesity, abdominal obesity, and metabolic syndrome in seven Latin American cities: the CARMELA Study. Eur J Cardiovasc Prev Rehabil.

[54] Monteiro CA, Conde WL, Lu B, Popkin BM (2004). Obesity and inequities in health in the developing world. Int J Obes.

[55] Hejazi N, Rajikan R, Choong CLK, Sahar S (2013). Metabolic abnormalities in adult HIV infected population on antiretroviral medication in Malaysia: a cross-sectional survey. BMC Public Health.

[56] Adewole O, Eze S, Betiku Y, Anteyi E, Wada I, Ajuwon Z, Erhabor G (2010). Lipid profile in HIV/AIDS patients in Nigeria. Afr Health Sci..

[57] Samaras K, Wand H, Law M, Emery S, Cooper D, Carr A (2007). Prevalence of metabolic syndrome in HIV-infected patients receiving highly active antiretroviral therapy using International Diabetes Foundation and Adult Treatment Panel III criteria: associations with insulin resistance, disturbed body fat compartmentalization, elevated C-reactive protein, and hypoadiponectinemia. Diabetes Care.

[58] Chehade JM, Gladysz M, Mooradian AD (2013). Dyslipidemia in type 2 diabetes: prevalence, pathophysiology, and management. Drugs.

